# Metal Organic Frameworks Modified Proton Exchange Membranes for Fuel Cells

**DOI:** 10.3389/fchem.2020.00694

**Published:** 2020-08-11

**Authors:** Quanyi Liu, Zekun Li, Donghui Wang, Zhifa Li, Xiaoliang Peng, Chuanbang Liu, Penglun Zheng

**Affiliations:** College of Civil Aviation Safety Engineering, Civil Aviation Flight University of China, Guanghan, China

**Keywords:** metal-organic framework, proton exchange membrane, modification, proton conductivity, fuel cells

## Abstract

Proton exchange membrane fuel cells (PEMFCs) have received considerable interest due to their low operating temperature and high energy conversion rate. However, their practical implement suffers from significant performance challenge. In particular, proton exchange membrane (PEM) as the core component of PEMFCs, have shown a strong correlation between its properties (e.g., proton conductivity, dimensional stability) and the performance of fuel cells. Metal-organic frameworks (MOFs) as porous inorganic-organic hybrid materials have attracted extensive attention in gas storage, gas separation and reaction catalysis. Recently, the MOFs-modified PEMs have shown outstanding performance, which have great merit in commercial application. This manuscript presents an overview of the recent progress in the modification of PEMs with MOFs, with a special focus on the modification mechanism of MOFs on the properties of composite membranes. The characteristics of different types of MOFs in modified application were summarized.

## Introduction

**Figure 1:**
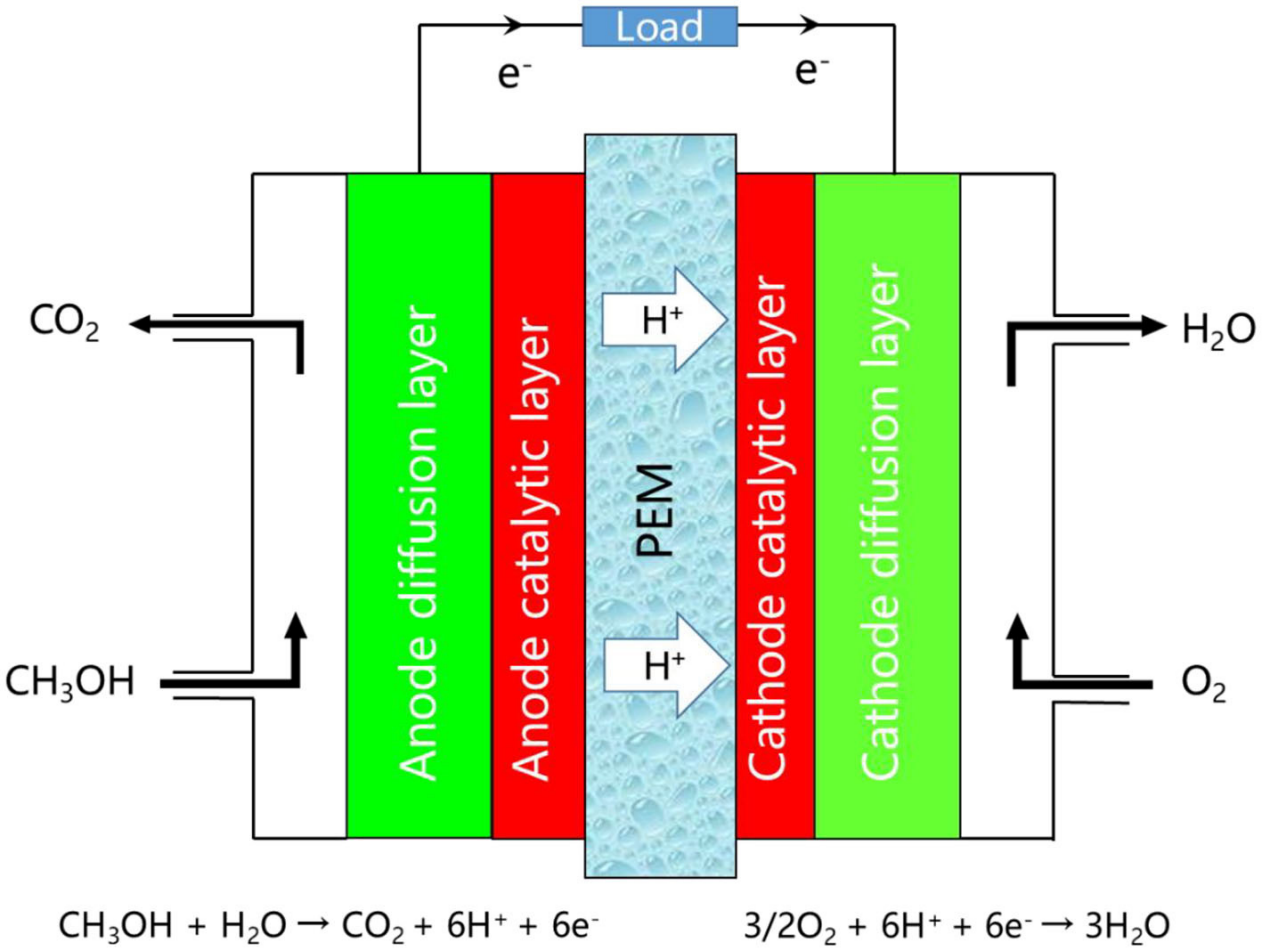
Working principle of DEMFC.

The shortage of fossil energy and the promotion of environmental protection concept have motivated the development of proton exchange membrane fuel cells (PEMFCs). PEMFCs were first developed by General Electric in the United States in the 1960s for the Gemini space program (Zhao et al., 2017; Teixeira et al., 2020). Compared with the other fuel cells (alkaline fuel cells, phosphoric acid fuel cells, molten carbonate fuel cells, etc.), PEMFCs possess several favorable features, including low operating temperature, fast start-up, high energy conversion rate, and environmentally friendliness (Moreno et al., 2015; Dekel, 2018; Pourrahmani et al., 2019; Zhang et al., 2019). PEMFCs are mainly composed of seal rings, collector plates and membrane electrode assemblies (MEAs) (Bose et al., 2014; Sanda et al., 2015; Li et al., 2020; Yang et al., 2020). MEAs convert the chemical energy of reactants into electrical energy, and directly determines the performance of the entire fuel cell system. Generally, MEAs consist of PEMs, catalytic layers and gas diffusion layers on both sides. Taking the direct methanol fuel cell (DEMFC) as an example, methanol reaches the anode catalytic layer through the gas diffusion layer, and the oxidation reaction takes place on the catalyst surface to generate electrons, protons and carbon dioxide (Jing et al., 2020; Junoh et al., 2020). Carbon dioxide is discharged through the anode outlet, and protons are transferred to the cathode catalyst layer through the PEM. At the same time, electrons are collected by the anode collector plates, and transferred to the cathode through the external circuit to form a current, which combines with protons and oxygens in the cathode catalyst layer to generate water (Rivera-Gavidia et al., 2020; Yan et al., 2020). The working principle of DEMFC is shown in Figure 1.

In recent years, remarkable achievements have been made in the research of catalyst and water management (Lv et al., 2016; Deng et al., 2019a, 2020). Many researchers have prompted extensive investigated PEM due to it's the key component of MEAs (Yuan et al., 2020). As the carrier of proton transfer, the PEM transfers protons produced by the anode catalytic layer to the cathode catalytic layer and react with oxygen to generate water. At the same time, the PEM as a physical barrier separates the anode fuel from the cathode fuel to avoid direct contact between them. In addition, PEM have not conduct electrons, forcing electrons to conduct through external circuits to generate current (Han and Wu, 2018; Zhao et al., 2019). At present, the most widely used PEMs is the Nafion membrane of DuPont Company. However, due to the high price of Nafion membrane, scientists are actively seeking alternative materials to Nafion membrane, such as sulfonated poly (aryl ether)s (Hooshyari et al., 2020). The introduction of sulfonic acid group can obviously improve the conductivity of membranes. Nevertheless, these materials will appear serious swelling with the increase of sulfonation degree, which greatly reduces the dimensional stability, and aggravate the penetration of fuel. Moreover, these alternative materials and Nafion membrane have the disadvantage of relying excessively on moisture content to maintain stable performance. Currently, a wide range of strategies have been proposed including grafting, cross-link, polyelectrolyte modification and adding functional particles in the PEMs (Koros and Zhang, 2017; Zhang et al., 2019).

As a kind of inorganic-organic hybrid material with a certain crystal structure, MOFs are mainly used in gas storage, gas separation, magnetic materials and reaction catalysis (Makiura et al., 2010; Shah et al., 2013; Knebel et al., 2018; Luo et al., 2018; Deng et al., 2019b; Feng et al., 2019). In recent years, modification of PEMs with metal-organic frameworks (MOFs) has been intensively studied (Furukawa et al., 2014; Shiqiang et al., 2016; Luo et al., 2017; Shaari et al., 2018; Cai et al., 2019; Deng et al., 2019c; Wang et al., 2019). It is found that the open framework structure of MOFs improved the performance of the composite membrane by loading particles with special functions into the matrix as proton carriers (Pardo et al., 2011; Dybtsev et al., 2014; Díaz-Duran and Roncaroli, 2017; Liu et al., 2018). The large specific surface area of MOFs allows the composite membrane to promote the migration of protons while accommodating more bound water, which further improves the proton hopping conductivity. In addition, the small pore size of MOFs can hinder fuel and oxidant diffusion to improve selectivity, and most MOFs themselves have many proton hopping sites, which can elevate the conductivity (Matoga et al., 2015; Ono et al., 2017; Wu et al., 2017; Luo et al., 2019).

This review focus on the recent advances of a few MOFs modified PEMs, including UiO-series, MIL-series, ZIF-series and others. Especially, the effects of different series of MOFs materials on the electrical and mechanical properties of PEMs are extensively summarized. To conclude, the challenges and perspectives in this field are put forward. We believe this review can provide a progress report for the research of MOFs modified PEM, and provide some inductive assistance for researchers.

## MOFs-Modified PEMs

Recently, MOFs with the high proton conductivity have received extensive attention. It was found that protons could be delivered through the coordination skeleton itself (Ohkoshi et al., 2010) or carriers (Bureekaew et al., 2009). However, the grain boundary structure of MOFs restricts the migration of proton conductors, resulting in insufficient conductivity. Furthermore, it is very difficult to directly process MOFs for fuel cells due to their special and diverse crystal structures (Taylor et al., 2015; Yang et al., 2015b; Pili et al., 2016; Van Goethem et al., 2018). Hybridization of MOFs with other polymers to form composite membranes is an effective strategy to solve this plight (Shekhah et al., 2011; Denny and Cohen, 2015; Kitao et al., 2017).

The PEMs modified by MOFs can be generally divided into two approaches. One of the approaches is to immerse the pores of MOFs with different proton carries, such as phytic@MIL, PIL@MIL, acids@MIL (Li et al., 2014a, 2016; Dong et al., 2017). Another approach is to modify the organic ligands of MOFs with functional groups (-SO_3_H, -NH_2_,−2COOH) to enhance the acidity and hydrophilicity. However, there are few reports on modified PEMs in different MOF categories. This section will summarize the performance improvement of PEMs with different categories of MOFs, include UiO-series, MIL-series, ZIF-series and other MOFs.

### UiO-Series MOFs PEMs

UiO-series MOFs (UiO for University of Oslo) are composed of an inner Zr_6_O_4_(OH)_4_ core bounded to twelve terephthalate ligands (Cavka et al., 2008; Kandiah et al., 2010). They exhibited exceptional chemical and thermal stabilities which was benefiting from the highly oxyphilic nature of Zr(IV) atoms and their strong coordination bonds with carboxylate oxygen (Mukhopadhyay et al., 2019; Zeng et al., 2019). Among them, the unique spatial structure of UiO-66 endows with good stability for a wide class of solvents such as water, acidic conditions, high temperature and pressure. In addition, UiO-66 has attracted considerable interests in modifying PEMs due to their high working capacity, excellent thermal stability, and low-cost regenerability (Devautour-Vinot et al., 2012; Katz et al., 2013; Wu et al., 2013b; Liu et al., 2015). The conductivity of the PEMs by adding UiO-66 was increased by 10–50%, and the penetration of fuel was decreased significantly. Moreover, the different linkers of UiO-66 can be used to tailor the affinity with the polymer matrix, which was conducive to the preparation of composite PEMs by blending with polymer matrix (He et al., 2017). Table 1 lists some typical PEMs modified with UiO-series MOFs.

**Table 1:** Properties of UiO-series MOF modified membranes.

**Compound**	**σ (S cm^**−1**^)**	**E_**a**_ (eV)**	**Conditions**	**References**
Nafion/UiO-66	1.65 × 10^−1^	n/a	80^o^C, 95% RH	Donnadio et al., 2017
Nafion/UiO-66-SO_3_H	1.71 × 10^−1^	n/a	80^o^C, 95% RH	Donnadio et al., 2017
Nafion/UiO-66-NH_2_	1.84 × 10^−1^	n/a	80^o^C, 95% RH	Rao et al., 2017b
Nafion/UiO-66-NH_2_+UiO-66-SO_3_H	2.56 × 10^−1^	n/a	90^o^C, 95% RH	Rao et al., 2017b
CS/UiO-66-NH_2_+UiO-66-SO_3_H	5.2 × 10^−2^	0.131	100^o^C, 98% RH	Dong et al., 2018
SPEEK/GO@UiO-66-SO_3_H	2.68 × 10^−1^	0.093	70^o^C, 95% RH	Sun et al., 2017a
Nafion/GO@UiO-66-NH_2_	3.03 × 10^−1^	n/a	90^o^C, 95% RH	Rao et al., 2017a
Nafion/PWA@UiO-66-NH_2_	9.2 × 10^−2^	n/a	25^o^C	Yang et al., 2019

Nafion membrane has become the most widely used PEMs by virtue of its high performance, and plays a significant role in the advancement of PEMFCs technology. The molecular structure of Nafion membrane is composed of hydrophobic poly tetra fluoroethylene main chain and perfluoroether branch chain with the hydrophilic sulfonic acid group at the end. This unique type of structure imparts Nafion membrane excellent chemical and thermal stability and favorable proton conductivity (Shao et al., 2007). However, the performance will be seriously reduced at low humidity, while sufficient humidification is necessary for superior performance. Compared with the pristine Nafion membrane, the proton conductivity of the composite membrane prepared by adding UiO-66 as filler into Nafion matrix was obviously improved by 30% under 95% RH (Donnadio et al., 2017). Research showed that the improvement of conductivity had no linear relationship with a load of MOFs fillers. The reason may be attributed to the excessive addition of low conductivity fillers that will offset the positive influence of fillers on the transport properties of ionomers. And the internal reunion of MOF fillers could cause resistance to conductivity (Khdhayyer et al., 2017; Lin et al., 2018). Consequently, the fillers should be added within a reasonable range, otherwise the adverse situation may occur.

**Figure 2:**
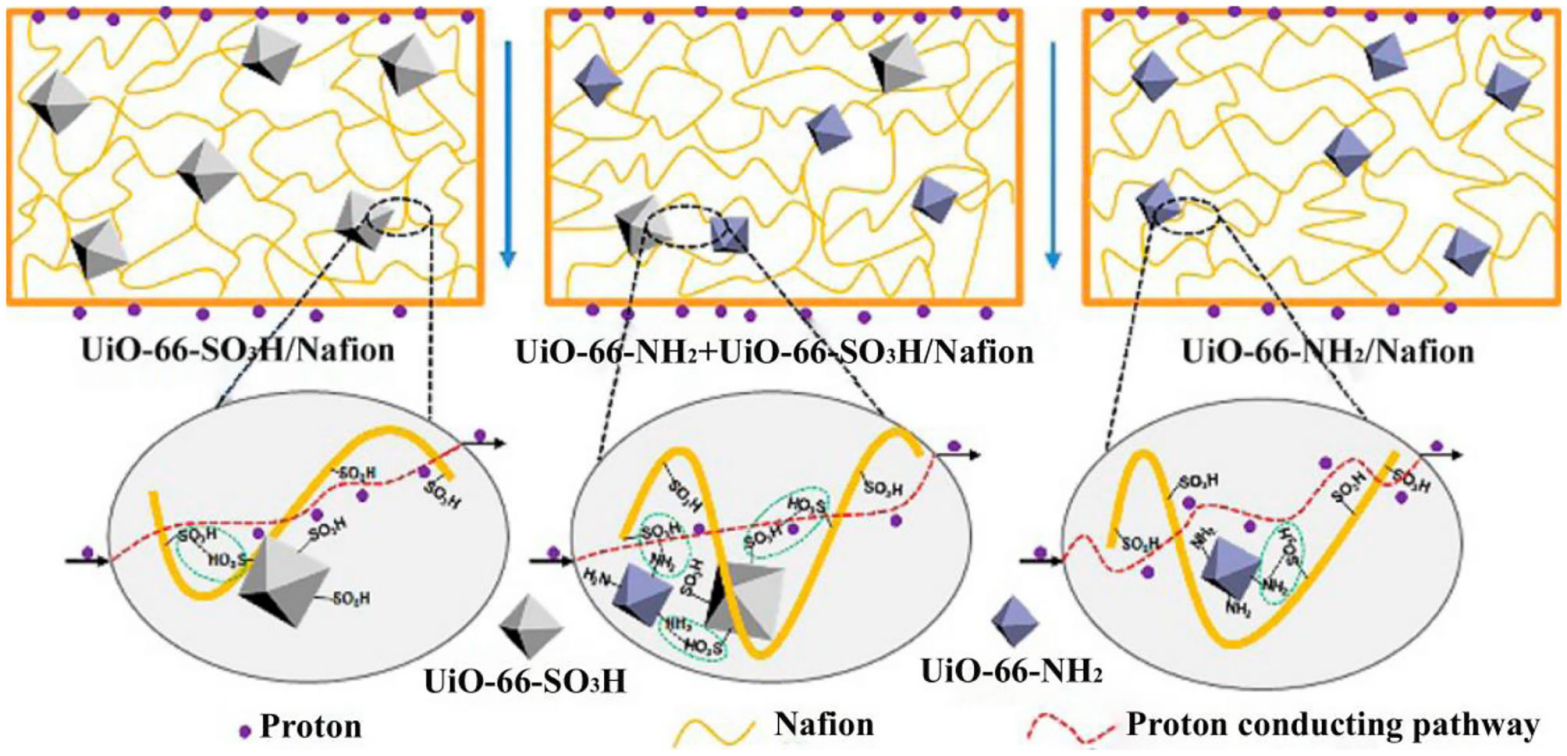
Transport mechanism diagram of Nafion/UiO-66-NH_2_, Nafion/UiO-66-SO_3_H, and Nafion/UiO-66-NH_2_+UiO-66-SO_3_H. [Readapted with permission from Rao et al. (2017b) Copyright 2017, American Chemical Society].

It was reported that UiO-66–SO_3_H possesses super protonic conductivity, numerous scholars have studied it as the main object of modified PEMs. Relative to the low proton conductivity of UiO-66 about 7 × 10^−6^ S cm^−1^, the proton conductivity of sulfonated UiO-66 has significantly increased by four orders of magnitude (Yang et al., 2011; Matthew et al., 2016; Patel et al., 2016). The sulfonic acid groups of UiO-66–SO_3_H are easily combined with water molecules, which could provide additional proton transfer pathways and more proton conduction sites. The proton conductivity of Nafion/UiO-66–SO_3_H was twice that of pristine Nafion membrane under the same conditions. The thermal stability of composite membrane was also slightly improved. Similar to the sulfonic acid groups, the amino groups can be used as proton carrier sites to improve proton conductivity (Ru et al., 2018). The composite membrane was synthesized by codoping of UiO−66-SO_3_H and UiO-66-NH_2._ It was found that the synergistic effect between UiO−66-SO_3_H and UiO-66-NH_2_ efficiently enhanced the proton conductivity of composite PEMs (Figure 2).

Inspired by this, UiO-66-SO_3_H and UiO-66-NH_2_ were codoped in chitosan (CS) polymer to synthesize proton-conducting hybrid membranes (Dong et al., 2018). The novel dual-MOF-cofilled hybrid membranes could work in both hydrated and anhydrous conditions. Surprisingly, the maximum proton conductivity of the dual-MOF-cofilled hybrid membranes obtained 3.78 × 10^−3^ S cm^−1^ under anhydrous conditions at 120°C, which was roughly 29.1 times as the pure CS membrane. In addition, a PEMFC was constructed by using CS/UiO-66-SO_3_H+UiO-66-NH_2_ as a membrane, which gave an open-circuit voltage of 1.0 V, and power density of 10.6 mW cm^−2^. The experimental results showed that the cause may be: (1) the synergy between acid groups (–SO_3_H) and amino groups (-NH_2_) reduced the barrier of proton conduction and formed a hydrogen network, leading to an increase in proton conduction channels; (2) sulfonate functional groups of UiO-66-SO_3_H as proton donors increase the number of proton carriers and enhanced high-temperature resistance of dual-MOFs-cofilled hybrid membranes; (3) -NH_2_ groups of the embedded basic MOFs as proton acceptor provide proton hopping sites and build efficient acid-basic pairs between those adjacent MOFs (Yang et al., 2016).

**Figure 3:**
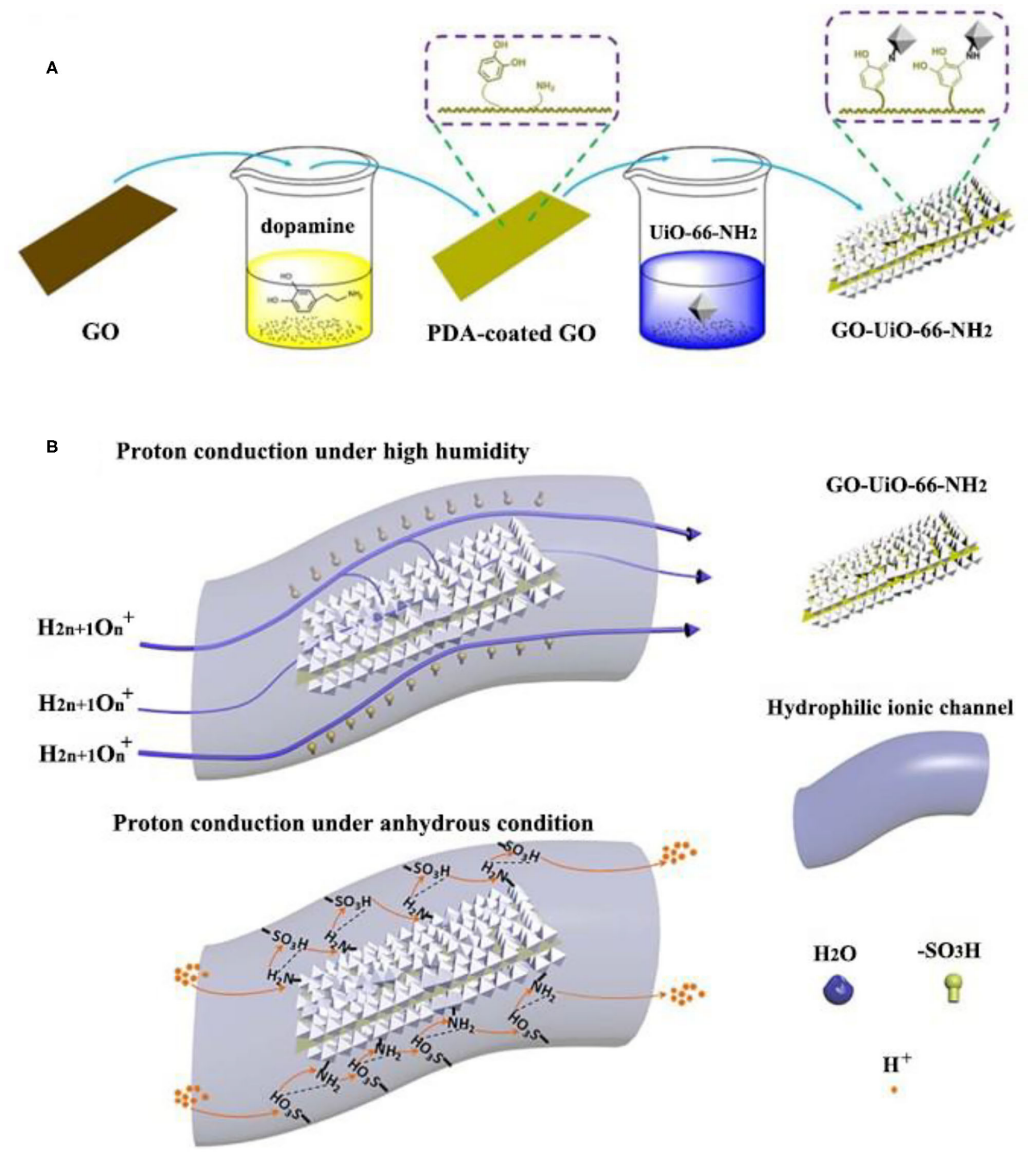
**(A)** Preparation of GO@UiO-66-NH_2_. **(B)** Schematic diagram of proton conductivity enhancements of GO@UiO-66-NH_2_. [Readapted with permission from Rao et al. (2017a) Copyright 2017, Elsevier].

Graphene oxide (GO) has good proton conductivity due to high surface area and numerous oxygen-containing functional groups. Moreover, a large number of carboxyl groups of GO coordinate with Zr ions of UiO-66, which promote the uniform growth of UiO-66 on the surface of GO. GO@UiO-66-NH_2_ has been successfully prepared (Figure 3A) and incorporated to the Nafion matrix to fabricate Nafion/GO@UiO-66-NH_2_ mixed membrane (Rao et al., 2017a,b). Particularly, under 95% RH and anhydrous conditions, the highest proton conductivity of the composite membrane was 0.303 S cm^−1^ and 3.4 × 10^−3^ S cm^−1^ at 90°C, which were 1.57 times and 1.88 times higher than that of recast Nafion, respectively. The principle that GO@UiO-66-NH_2_ improves proton conduction can be clearly seen from Figure 3B. The reasons for improving proton conduction are speculated as follows: (1) massive of oxygen-containing functional groups in GO act as proton receptors; (2) excellent water affinity and high specific surface area of UiO-66-NH_2_ enhanced the water retention capacity of PEMs; (3) the continuous proton transport channels of UiO-66-NH_2_ was constructed by the tethering effect of GO surfaces and the good interaction between MOF particles; (4) the synergy between acid groups (–SO_3_H) of Nafion and basic groups (–NH_2_) of MOF provided extra proton hopping sites, and build efficient acid-basic pairs between those adjacent MOFs. In addition, the barrier effect of GO increased the tortuosity of transport channels and UiO-66-NH_2_ could trap methanol inside its pores, thus ameliorated the permeation of methanol.

**Figure 4:**
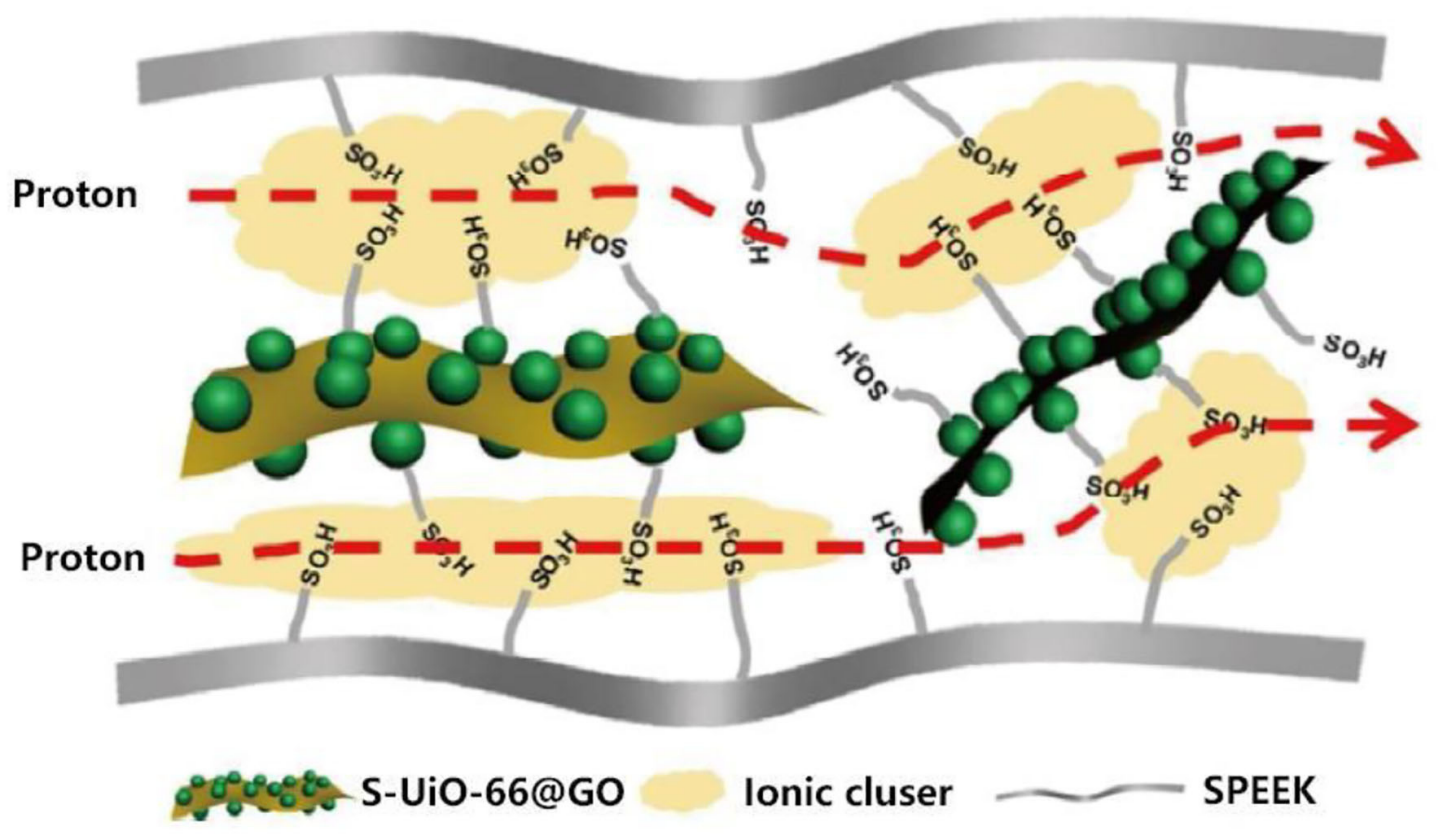
Schematic illustration of the enhanced transport properties of the GO@UiO-66-SO_3_H. [Readapted with permission from Sun et al. (2017a) Copyright 2017, American Chemical Society].

Sun et al. prepared GO@UiO-66-SO_3_H hybrid nanosheets via *in situ* surface growth method, and incorporated them into sulfonated polyether ether ketone (SPEEK) to synthesized SPEEK/GO@UiO-66-SO_3_H composite membranes (Yang et al., 2011; Sun et al., 2017a). Proton can be transported in the ionic nanochannels formed by the assembly of -SO_3_H groups of GO@UiO-66-SO_3_H together with -SO_3_H groups of SPEEK (as shown in Figure 4). The *in-situ* surface growth method effectively eliminated the agglomeration of UiO-66-SO_3_H, and improve proton conductivity of composite membranes. The effect of different functional groups (i.e., –SO_3_H, −2COOH, –NH_2_, and –Br) on proton conductivity of UiO-66 was investigated (Yang et al., 2015a). The results revealed that the proton conductivity of UiO-66-SO_3_H and UiO-66-2COOH were three orders of magnitude higher than pure UiO-66. Therefore, the –SO_3_H and −2COOH had great application potential in modifying the PEMs.

Phosphotungstic acid has been coupled with modified UiO-66-NH_2_ and incorporated into the Nafion membrane by solution casting to improve the proton conductivity and ion-selectivity (Garibay and Cohen, 2010; Yang et al., 2019). The composite membrane exhibited a higher proton conductivity (9.2 × 10^−2^ S cm^−1^) and an ultra-high ion-selectivity (2.66 × 10^5^ S min cm^−3^). In many cases, the performance of doping pure MOFs in PEMs was unsatisfactory. The effect of combining nanoparticles with MOFs was incredible to improve the performance composite membranes.

### MIL-Series MOFs PEM

MIL-series MOFs (MIL for Materials of Institute Lavoisier) are synthesized by different transition metals and dicarboxylic ligands such as succinic acid and glutaric acid. Dicarboxylic ligands have been introduced many functional groups through appropriate chemical modifications. Compared with the traditional zeolite materials and graphite materials, MIL-series MOFs have the unique advantages of porosity, large specific surface area and unsaturated metal coordination sites. For instance, MIL-100 is a crystal with special topological structure synthesized by trivalent chromium and pyromellitic acid, that its pore diameter reaches 2.5–2.9 nm. MIL-101 with 3.0–3.4 nm of pore size and 5,900 m^2^/g of specific surface area is synthesized from trivalent chromium octahedral clusters and terephthalic acid. MIL-101 has two types of cage structure limited by 12 pentagonal faces for the smaller and by 16 faces for the larger. The proton conductivity of composite membranes can be obviously improved by adding MIL-series MOFs. The large number of coordinatively unsaturated metal sites (CUSs) of MIL-101 can provide abundant -OH group through hydrolysis. These -OH groups can form hydrogen-bond networks in the proton exchange membrane, which promote proton conduction through the Grotthuss mechanism. MIL-53 (Al) is connected with terephthalic acid group by aluminum as the connection point of metal organic framework, formed one-dimensional channels with a diameter of about 8.5 Å. Water molecules can be trapped by the hydrogen-bonding of carboxylic acid groups in these channels, which improve the conductivity of protons (Amdursky et al., 2016). In recent years, the research on the application of MIL-series MOFs has attracted wide attention from researchers.

Five types of MOFs were prepared (MIL-101, MIL-101-SO_3_H, H_2_SO_4_@MIL-101, H_3_PO_4_@MIL-101, CF_3_SO_3_H@MIL-101), and various hybrid membranes were synthesized via the solution casting method (Dong et al., 2017). The highest proton conductivity of pure CS membrane and hybrid membranes and other characteristic parameters are shown in Table 2. It is apparent that the incorporation of MIL-101 has increased proton conductivity by 2~3 times. The analysis indicated that the internal hydrogen-bond network interaction and the electrostatic effect can be contributed to the significant improvement of the proton conductivity (Hupp and Poeppelmeier, 2005). Furthermore, these non-volatile acids are more compatible with MOFs, which provide additional transport receptors and hopping sites while further enhancing the proton conductivity of the composite membranes.

**Table 2:** The glass transition temperatures (T_d_), proton-conducting activation energy (E_a_), and proton conductivity of hybrid membranes.

**Hybrid membranes**	**T_**d**_ (**°**C)**	**E_**a**_ (eV)**	**σ (S cm^**−1**^)**	**References**
CS	217.7	0.189	0.030	Dong et al., 2018
CS/MIL-101	226.7	0.183	0.034	Dong et al., 2017
CS/S-MIL-101	234.3	0.174	0.064	Dong et al., 2017
CS/H_2_SO_4_@MIL-101	220.1	0.181	0.095	Dong et al., 2017
CS/H_3_PO_4_@MIL-101	226.8	0.175	0.083	Dong et al., 2017
CS/CF_3_SO_3_H@MIL-101	230.8	0.179	0.094	Dong et al., 2017

Similar work has been done (Li et al., 2014b) to study the effect of sulfonic acid groups on the proton conductivity of composite membranes. They fabricated a new kind of hybrid membrane by incorporated sulfonated MIL-101(Cr) into the SPEEK matrix. The protons conductivity of SPEEK/sul-MIL-101(Cr) was increased up to 0.306 S cm ^−1^, which was approximately doubled as the SPEEK. Similar to the previous reasons, sulfonic acid groups as proton transition centers increased the number of proton channels while forming hydrogen-bonded networks through the hydrolysis of CUSs. Phytic acid (myo-inositol hexaphosphonic acid) and phosphotungstic acid are conducive to the conduction of protons, but there are few reports on the research of phytic acid and phosphotungstic acid. Phytic acid has the content of phosphoric acid group next to phosphoric acid while also having good conductivity, chemical stability, and chelating ability. Incorporation of the phytic@MIL-101 into the Nafion membrane significantly enhanced the conductivity in low relative humidities (Li et al., 2014a). The proton conductivity of hybrid membranes was surprisingly up to 7.63 × 10^−4^ S cm^−1^ at 10% RH, which was 11 times higher than the Nafion membrane. Phosphotungstic acid (HPW) were formed by incorporated Na_2_WO_4_·2H_2_O and Na_2_HPO_4_ into the cavities of MIL-101(Cr), which avoided the leakage of HPW due to its high water solubility. The SPEEK/HPW@MIL-101 membranes prepared with HPW@MIL-101 in SPEEK exhibited good proton conductivity at low relative humidity (7.25 times higher than pristine SPEEK membrane; Zhang et al., 2017).

**Figure 5:**
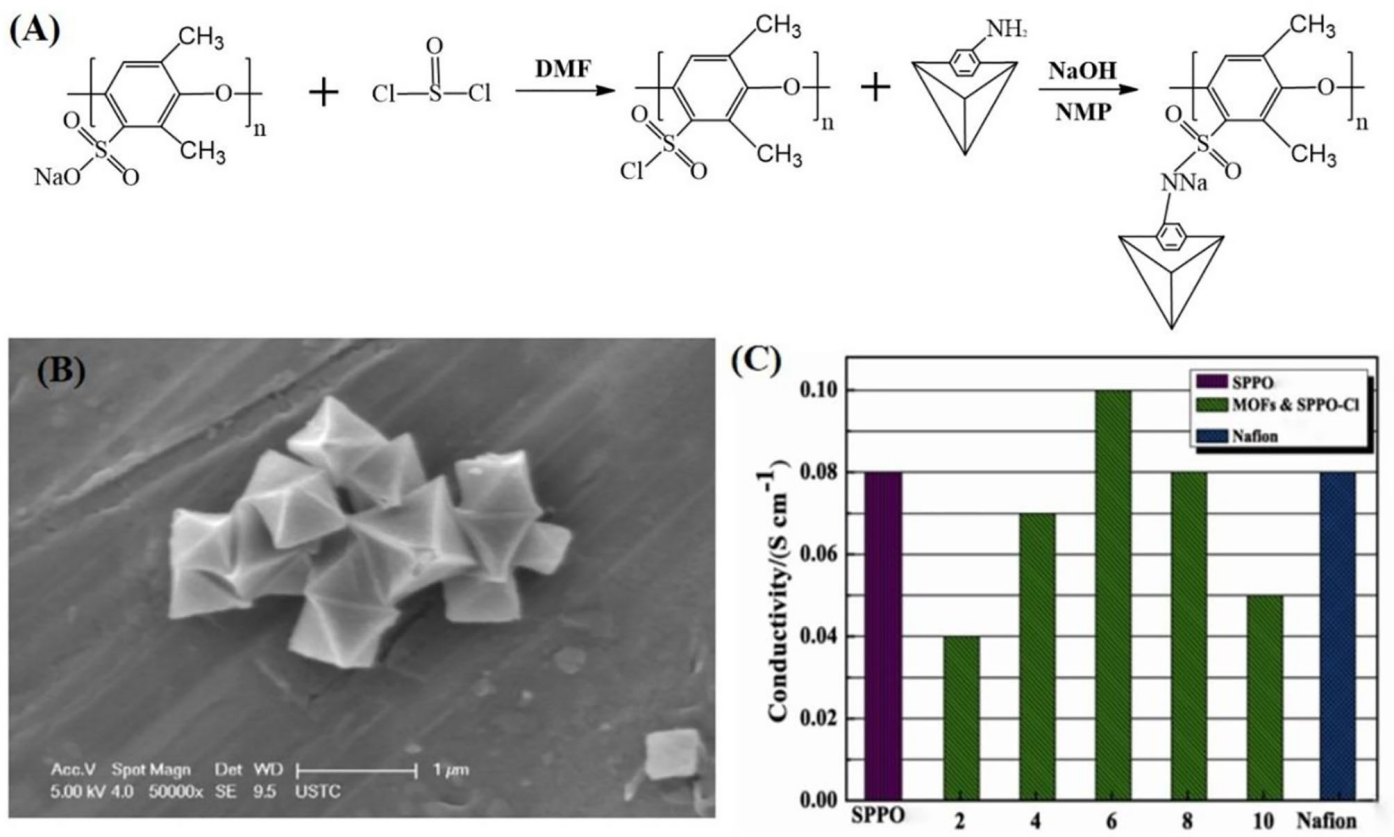
**(A)** The synthetic procedure for membranes of Fe-MIL-101-NH_2_-PPO–SO_2_Cl. **(B)** SEM images of Fe-MIL-101-NH_2_ microcrystals. **(C)** Proton conductivities of the hybrid membranes at different MOF loadings at room temperature. [Readapted with permission from Wu et al. (2013a) Copyright 2013, Royal Society of Chemistry].

**Figure 6:**
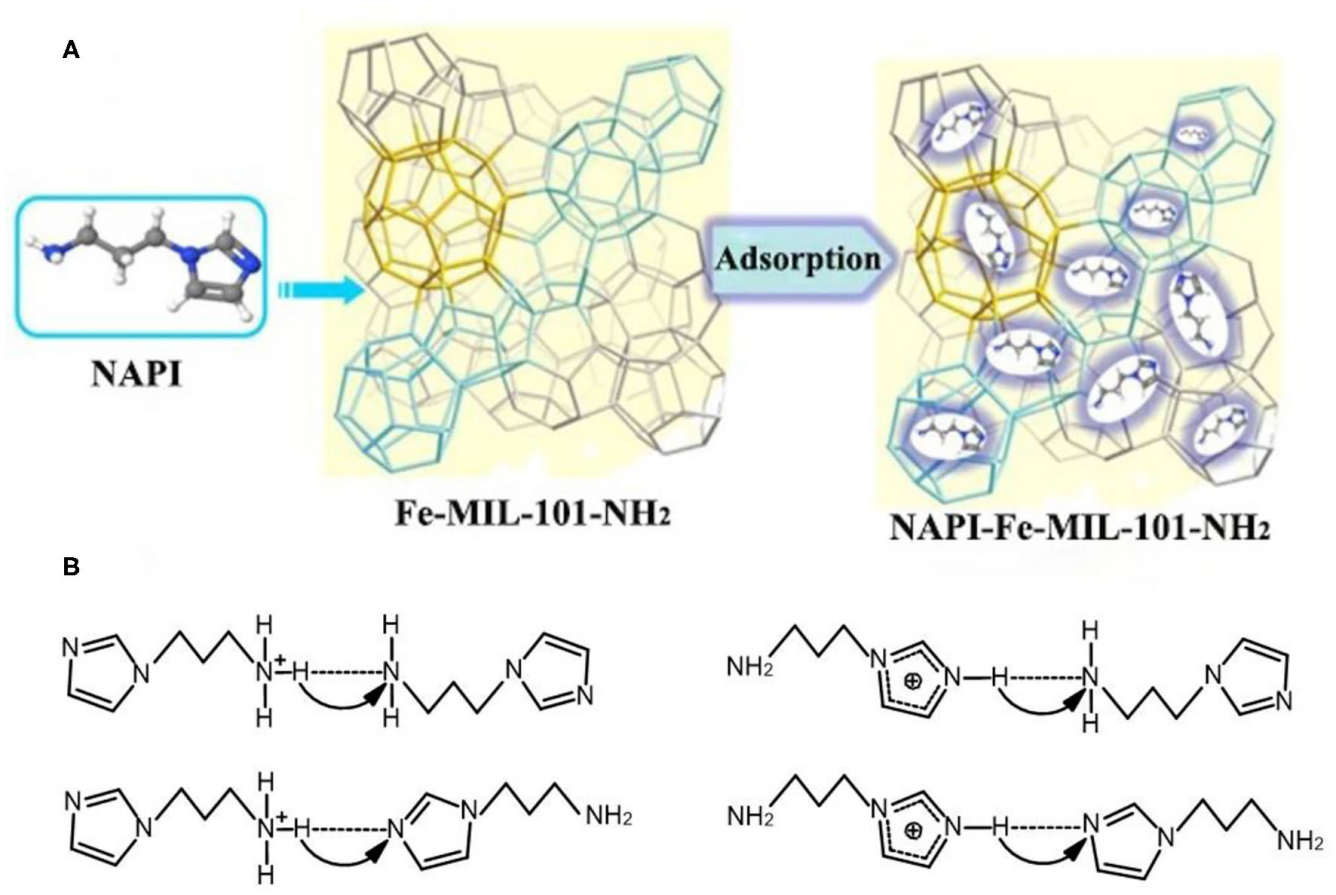
Schematic illustration of **(A)** NAPI adsorption into Fe-MIL-101-NH_2_. **(B)** intermolecular proton transfer in NAPI. [Readapted with permission from Wu et al. (2014a) Copyright 2014, Elsevier].

In addition to the above, amino functionalized MOFs is an effective strategy to improve conductivity of composite membranes (Anahidzade et al., 2018). A novel proton-conducting composite membrane was synthesized by amino-functionalized MIL-101 (Fe) particles with sulfonated poly (2, 6-dimethyl-1, 4-phenylene oxide) (SPPO) immersing in water and CHCl_3_ (Wu et al., 2013a). The procedure for preparing membranes with Fe-MIL-101-NH_2_-PPO–SO_2_Cl was shown in Figure 5A. Fe-MIL-101-NH_2_ particles were dispersed in matrix with almost no surface gap due to the chemical attachment between Fe-MIL-101-NH_2_ particles and SPPO matrix. The SEM showed that the Fe-MIL-101-NH_2_ crystals had an octagonal structure as presented in Figure 5B. Moreover, the additional protons were provided from the sulfonimide on a polymeric chain to further promote the proton conductivity. The proton conductivity of Fe-MIL-101-NH_2_-PPO–SO_2_Cl membrane reached to 0.10 S cm^−1^ at room temperature and 0.25 S cm^−1^ at 90°C, respectively, which was much higher than that of Nafion and pure SPPO membranes at the identical conditions (Figure 5C). On this basis, N-(3-aminopropyl)-imidazole (NAPI) was encapsulated in the frameworks of Fe-MIL-101-NH_2_, and mixed with SPPO to prepare composite PEM (Wu et al., 2014a). Liquid NAPI has much more proton acceptor/donor sites than imidazole, which ensured the continuous proton transfer by resided in the Fe-MIL-101-NH_2_ (Figure 6A). The schematic view of intermolecular proton transfer in NAPI as shown in Figure 6B. The composite membrane has maintained excellent proton conductivity and low methanol permeability at high temperature.

**Figure 7:**
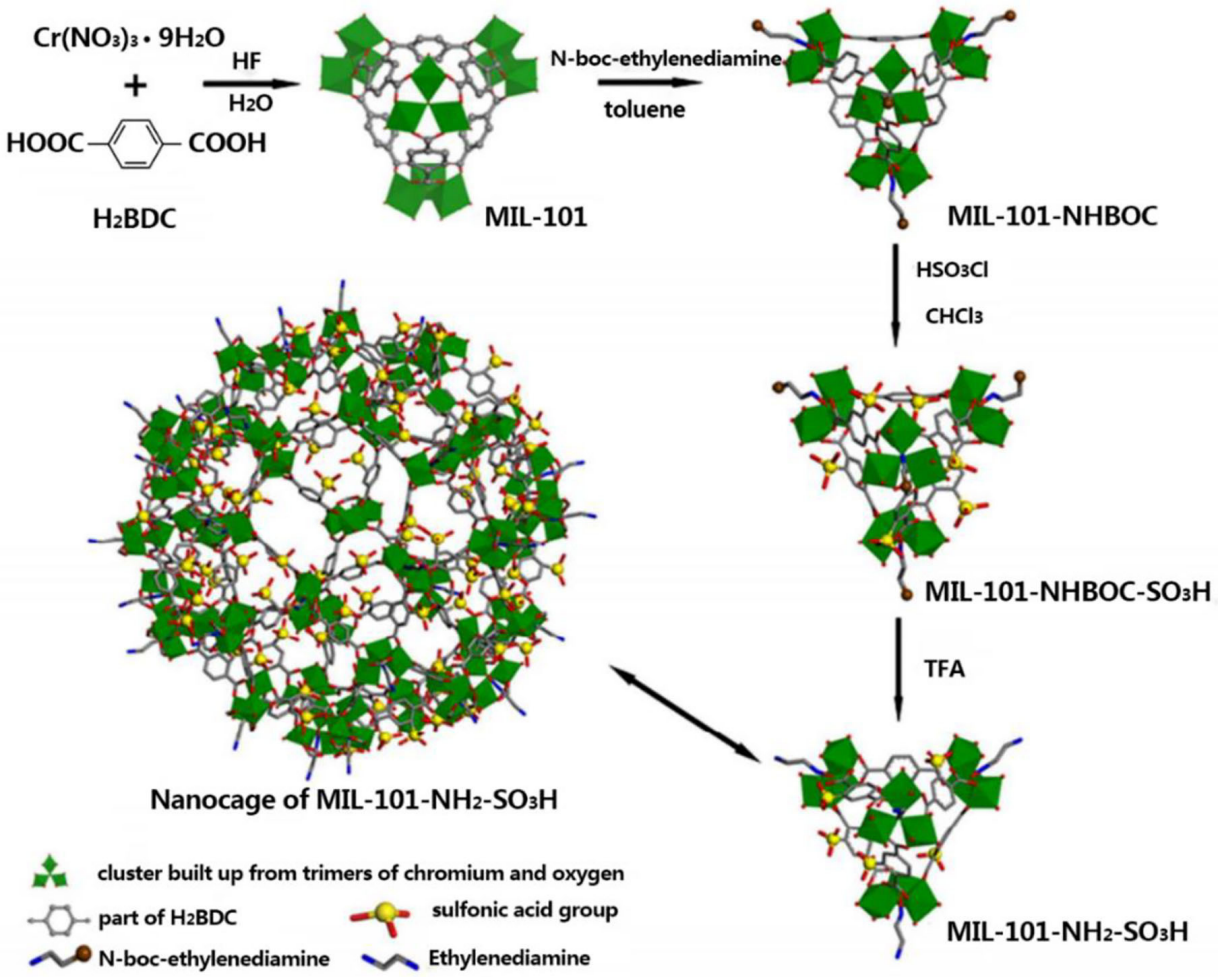
The schematic preparation and the nanostructure of MIL-101-NH_2_-SO_3_H. [Readapted with permission from Ru et al. (2018), Copyright 2018, American Chemical Society].

The sulfonic acid group could provide more proton conductive sites and transport pathways, and the amino group is the effective site for proton conduction. The combination of them greatly improve the conductivity of the composite membranes. A novel amino-sulfo-bifunctionalized MOFs named MIL-101-NH_2_-SO_3_H (MNS), was prepared and incorporated it into sulfonated poly (arylene ether ketone) containing naphthalene and fluorine moieties (SNF-PAEK) to form the MOFs-modified hybrid membranes (Ru et al., 2018). The schematic preparation and the nanostructure of MIL-101-NH_2_-SO_3_H are shown in Figure 7. The proton conductivity of these composite membranes was improved because MNS rearranges the microstructure of the membrane to accelerate the rate of proton migration.

1-(1-Ethyl-3-imidazolio)propane-3-sulfonate (EIMS) has been developed as another material suitable for proton conduction. A hybrid polymer proton-conducting material, EIMS-HTFSA@MIL, was fabricated by impregnating EIMS and *N,N*-bis(trifluoromethanesulfonyl) amide (HTFSA) into MIL-101 via heterogeneous mixing and grinding method. The protonated –SO_3_H/HTFSA in EIMS-HTFSA@MIL acted as the proton source and unprotonated –${\text{SO}}_{3}^{-}$/TFSA^−^ matrix as the proton defect sites, enabling hydrogen ions hopping between the adjacent –${\text{SO}}_{3}^{-}$ groups for efficient transport (Sun et al., 2017c). Remarkably, the proton conductivity of EIMS-HTFSA@MIL reached 2 × 10^−4^ S cm^−1^ at 140°C and the pyrolysis temperature was up to 320°C. It was showed that it has unlimited potential for application in high-temperature fuel cells. Extending this work, Chen Hui and co-workers investigated the acid counter anion (R-${\text{SO}}_{3}^{-}$) effect of PILs@MOF. They prepared various hybrid membranes, i.e., SA-EIMS@MIL-101, MSA-EIMS@MIL-101 and PTSA-EIMS@MIL-101 (SA = sulfate acid, MSA = methanesulfonate acid, PTSA = p-toluenesulfonate acid) by heterogeneously impregnating procedure (Chen et al., 2018). Compared to previous reported HTSFA, the R-${\text{SO}}_{3}^{-}$ has transfered protons more efficiently at low humidity due to their lower hydrophilicity. The van der Waals volumes of different acid counter anions are closely related to the proton conductivity of these hybrid membranes (Table 3). The steric hindrance inside the material became greater as the van der Waals volumes increased, which led to stronger blocking of hydrogen ions during transport, and made proton conductivity falling.

**Table 3:** Proton conductivities at 50°C and 150°C and activation energies of membranes.

**Hybrid membranes**	**σ** **(S cm**^****−1****^**)**		**E_**a**_ (eV)**	**V_**vdw**_(Å^**3**^)**	**References**
	**50**°**C**	**150**°**C**			
SA-EIMS@MIL-101	2.54 × 10^−4^	1.89 × 10^−3^	0.262	62.23	Chen et al., 2018
MSA-EIMS@MIL-101	8.46 × 10^−5^	1.02 × 10^−4^	0.304	70.74	Chen et al., 2018
PTSA-EIMS@MIL-101	7.67 × 10^−6^	2.78 × 10^−4^	0.424	143.36	Chen et al., 2018

Kitagawa et al. reported the crucial influence of coordinated water or guest molecules on proton conduction in MOFs (Sadakiyo et al., 2009, 2012; Yamada et al., 2009). On the basis, two porous MOFs [CPO-27(Mg) and MIL-53(Al)] were incorporated into Nafion membrane for the first time to enhance the water retention ability performance of the membrane (Loiseau et al., 2004; Dietzel et al., 2008; Tsai et al., 2014). The unique one-dimensional pore structure of CPO-27 (Mg) provides an extra place for water storage. Moreover, CPO-27(Mg) and MIL-53(Al) have accommodated the condensed water in the micro-pore of skeleton and produced intense interaction with guest water molecules. Thus, the water uptake and the proton transport efficiencies of the composite membranes were greatly improved by 1.7 and 2.1 times compared with the recast Nafion membrane, respectively. These results showed that MOFs with excellent water retention ability can greatly improve the conductivity of composite membranes and had great potential for application in PEMFCs.

### ZIF -Series MOFs PEM

Zeolite imidazolate frameworks (ZIFs), as a subclass of MOFs consist of a tetrahedral divalent metal cations CO^2+^ (or Zn^2+^) coordinated to four imidazolic acid rings with the metal–imidazolate–metal bond angles similar to the Si-O-Si angles of zeolites. The complete saturation of the metal provides an excellent thermal stability for ZIFs. In addition, the hydrophobic pores and surface structure of ZIFs are highly repulsion to water molecules, preventing the dissolution of the framework, giving ZIFs excellent chemical stability in alkaline aqueous solution, organic solvent and water. Several reports have revealed that the hybrid PEMs embedding ZIFs with a zeoltic topology possess superior stability and high proton conductivity (Park et al., 2006; Tan and Mahdi, 2016; Liu et al., 2020). The high microporosity and cavity volumes volume of ZIFs with a large number of proton carriers in the cavity enhances the conductivity of PEM. For instance, the unique structure of ZIF-8 with a large pores (11.6 Å in diameter) connected through small apertures (about 3.4 Å) have significantly reduced the penetration of methanol. The compatibility of ZIF-8 with the polymer matrix has a positive effect on the water management of composite membrane.

**Figure 8:**
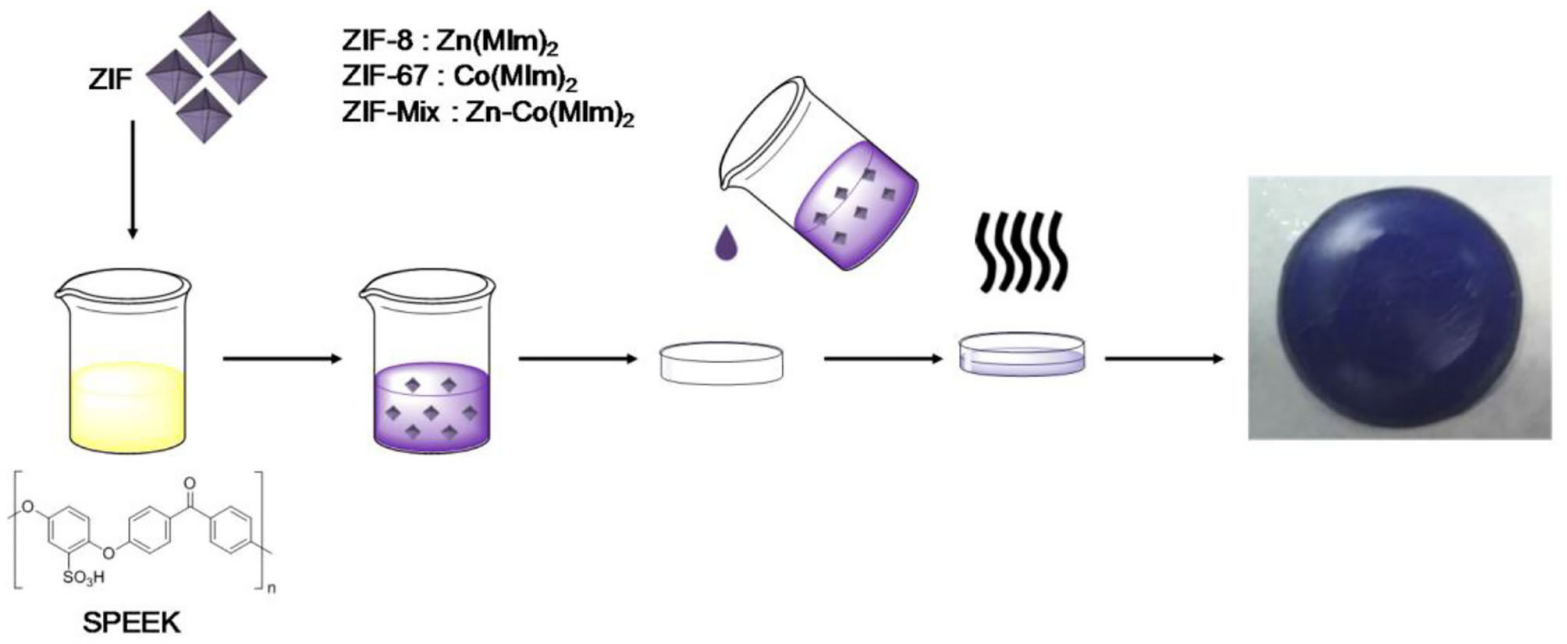
Schematic illustration of casting method for SPEEK@ZIFs membrane preparation. [Readapted with permission from Barjola et al. (2018), Copyright 2018, MDPI].

Based on the previous synthesis of PEI/ZIFs, Vicente Compañ and co-workers successfully prepared PBI@ZIF-8, PBI@ZIF-67, and PBI@ZIF-Mix (a Zn/Co bimetallic mixture) hybrid membranes (Shi et al., 2012; Vega et al., 2017; Escorihuela et al., 2018). Compared with the single ZIF doping, ZIF-mix doping is beneficial to improve the proton conductivity (Escorihuela et al., 2018). Moreover, Vicente Compañ et al. synthesized SPEEK@ZIF-8, SPEEK@ZIF-67, and SPEEK @ZIF-Mix hybrid membranes by similar methods (Figure 8; Barjola et al., 2018). The comparison between various membranes proton conductivity is shown in Table 4. The improvement of the conductivity can be attributed to the additional proton carriers provided from ZIFs.

**Table 4:** The proton conductivities of ZIF-series MOF modified membranes.

**Hybrid membranes**	**Additive**	**Conductivity (S cm^**−1**^)**	**Conditions**	**References**
PBI@ZIF-8	H_3_PO_4_	3.1 × 10^−3^	180^o^C, anhydrous	Escorihuela et al., 2018
PBI@ZIF-67	H_3_PO_4_	4.2 × 10^−2^	180^o^C, anhydrous	Escorihuela et al., 2018
PBI@ZIF-Mix	H_3_PO_4_	9.2 × 10^−2^	180^o^C, anhydrous	Escorihuela et al., 2018
SPEEK@ZIF-8	–	1.6 × 10^−2^	100^o^C	Barjola et al., 2018
SPEEK@ZIF-67	–	1.5 × 10^−2^	100^o^C	Barjola et al., 2018
SPEEK@ZIF-Mix	–	2.9 × 10^−2^	100^o^C	Barjola et al., 2018
PEI@ZIF-8	TBA	1.9 × 10^−3^	75^o^C, 95%RH	Vega et al., 2017
PEI@ZIF-67	TBA	1.8 × 10^−3^	75^o^C, 95%RH	Vega et al., 2017
PEI@ZIF-Mix	TBA	3.0 × 10^−3^	75^o^C, 95%RH	Vega et al., 2017
SPEEK@ZCN	CNTs	5.0 × 10^−2^	120^o^C, 30%RH	Sun et al., 2017b

**Figure 9:**
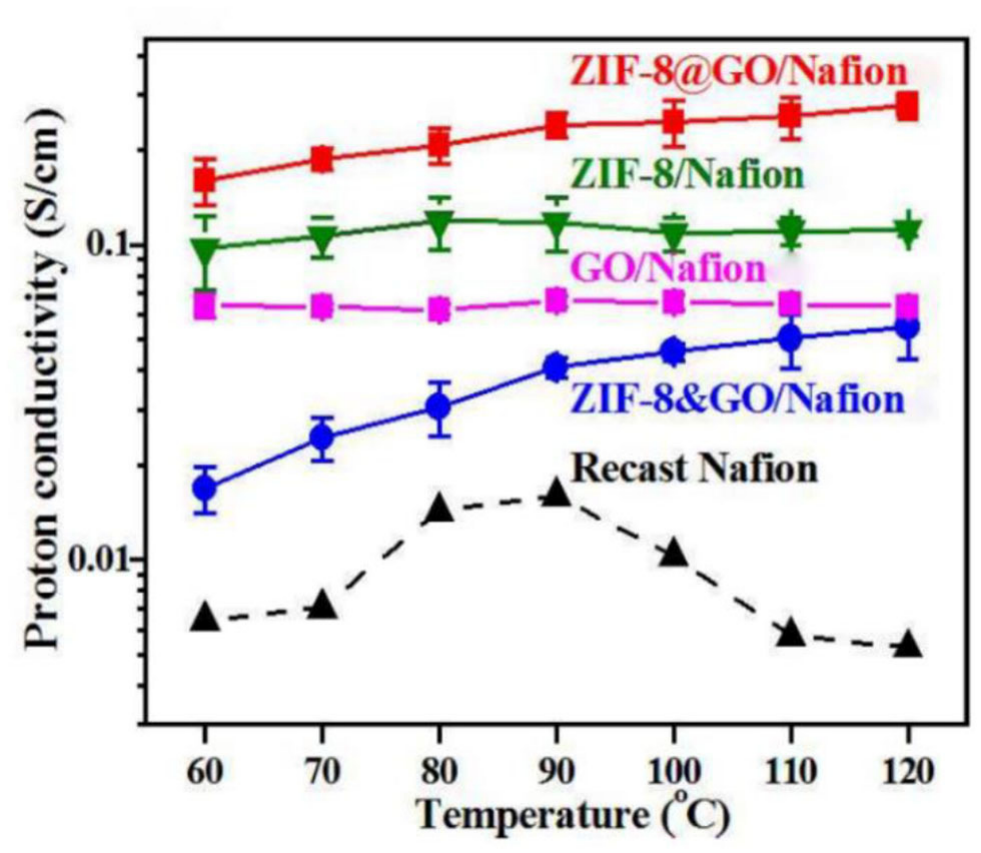
Pronton conductivity of ZIF-8@GO/Nafion and contrast membranes. [Readapted with permission from Yang et al. (2015b) Copyright 2015, Royal Society of Chemistry].

**Figure 10:**
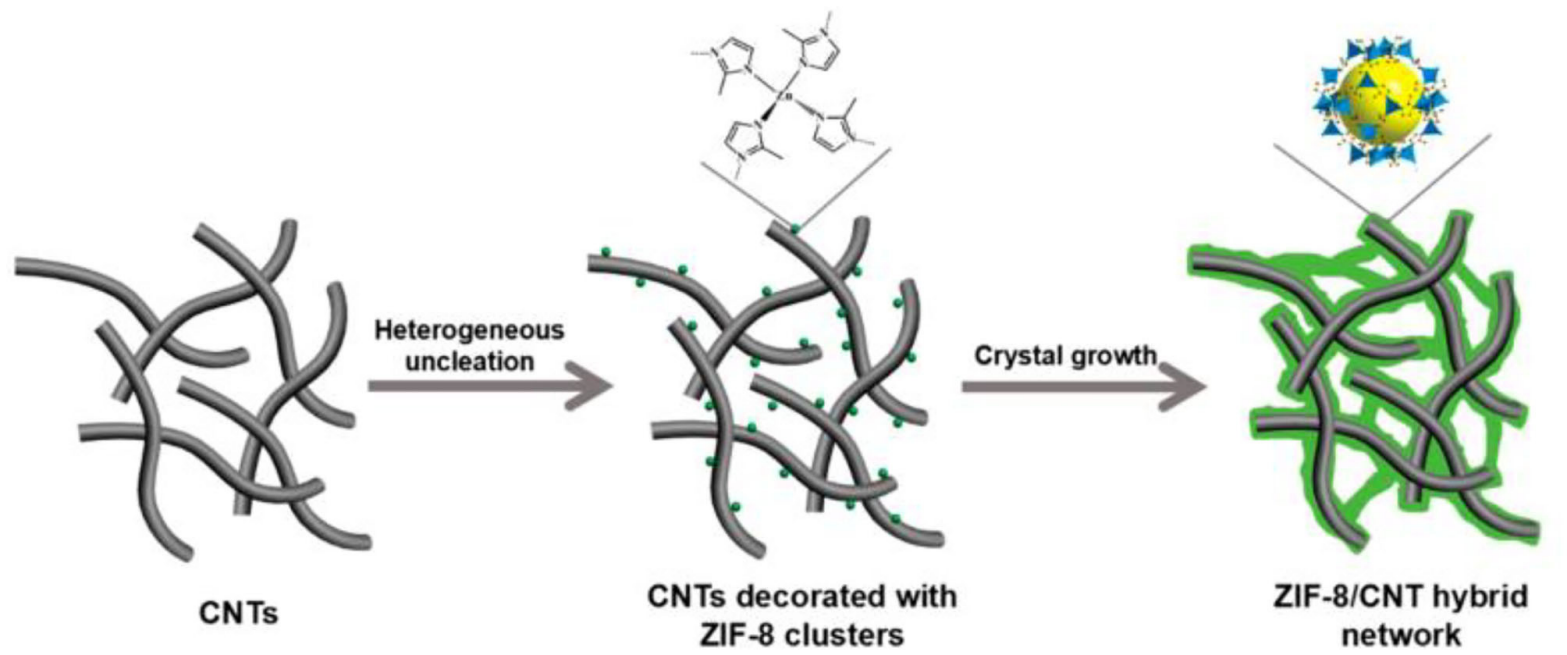
Schematic illustration of *in situ* growth method for ZIF-8/CNT synthesized. [Readapted with permission from Sun et al. (2017b) Copyright 2017, American Chemical Society].

The—N-H from the ZIF-8 framework can provide proton, and reduce the obstacles in the proton conduction path through interact with GO rich in oxygen-containing groups (Zhang et al., 2013). Figure 9 shows that the proton conductivity of the ZIF-8@GO/Nafion hybrid membrane was improved 55 times higher than the Nafion membrane at 120°C and 40% RH (Yang et al., 2015b). A novel two-dimensional zeolite structure ZIF-8/CNT hybrid crosslinked networks (ZCN) was successfully synthesized as shown in Figure 10. The introduction of ZCN and SPEEK significantly improved the proton conductivity and inhibited methanol permeability (Sun et al., 2017b). The proton conductivity of SPEEK@ZCN composite membrane was reaching 0.05 S cm^−1^ at 120°C and 30% RH, which is one of the highest ZIFs modified PEM so far. In addition, it was found that the addition of two-dimensional fillers significantly improved the proton conductivity of the membrane (Chen et al., 2016; Jia et al., 2016).

Poly(vinylphosphonic acid) (PVPA) has superior proton conductivity due to more phosphate groups and strong hydrogen bonding (Sen et al., 2016). However, the proton conductivity of PVPA decreases sharply at temperature above 100°C. The proton conductivity of PVPA can be improved by doping the ZIF-8 with high conductivity. The proton conductivity of ZIF-8@PVPA composite membrane has reached 3.2 × 10^−3^ S cm^−1^ at 140°C under anhydrous conditions, which was 3 times higher than that of pure PVPA membrane. In addition, the pyrolysis temperature of the composite membrane was increased to above 250°C. Poly(2-acrylamido-2-methyl-1-propanesulfonic acid) (PAMPS) is an acidic homopolymer with high proton conductivity. But it cannot be formed pristine membrane independently, which are greatly limited its application in fuel cells (Karlsson et al., 2002). The polymer composite membranes were prepared with poly(vinyl alcohol) (PVA), PAMPS and ZIF-8 in appropriate proportions, which overcome the shortcomings mentioned above (Erkartal et al., 2016).

Natural biomolecules can transport protons and ions through the synthesized transmembrane pores (Ordinario et al., 2014). Protein has been studied by many scholars as a typical proton conducting natural biomaterial (Nagle and Tristram-Nagle, 1983; Amdursky et al., 2016; Gopfrich et al., 2016). DNA@ZIF-8 membrane was prepared by incorporate single-strand DNA into the ZIF-8 membrane via a solid confinement conversion process (Guo et al., 2018). The composite membrane exhibited high proton conductivity of 0.17 S cm^−1^ at 75 °C, but low methanol permeability of 1.25 × 10^−8^ cm^2^ s^−1^ due to the smaller pore entrances size of ZIF-8. ZIF-8 has significant efficacy in solving ionic liquid leakage problems. Liu et al. fabricated an ionic liquids-polymer composite membrane with ZIF-8 fillers via the combination of the ionothermal and *in situ* crystallization method, which effectively inhibited the loss of ionic electrolyte (Liu et al., 2014).

### Other Kinds of MOFs Modified PEM

In the previous sections, a series of typical MOFs and composite membranes with satisfactory performance were introduced. In addition, there are some other PEMs with satisfactory properties, but they have the disadvantages that the synthesis is tedious and expensive. Therefore, we will give a brief introduction to these PEMs in this section.

An oriented electrospun nanofiber proton-conducting membrane was synthesized by compose Zn_2_(C_2_O_4_)(C_2_N_4_H_3_)_2_(H_2_O)_0.5_(ZCCH) and SPPEK. The composite membranes exhibited excellent proton conductivity (8.2 × 10^−2^ S cm^−1^) under high temperature and anhydrous conditions, while the methanol permeability is only about 6% of Nafion-115 under the same conditions (Wu et al., 2014b). A hexaphosphate ester-based 3D MOF named JUC-200 and its composite membrane with poly(vinyl alcohol) (PVA) were prepared (Cai et al., 2017). The composite membrane exhibited excellent water tolerance and high conductivity in a solution of PH = 2.0.

**Figure 11:**
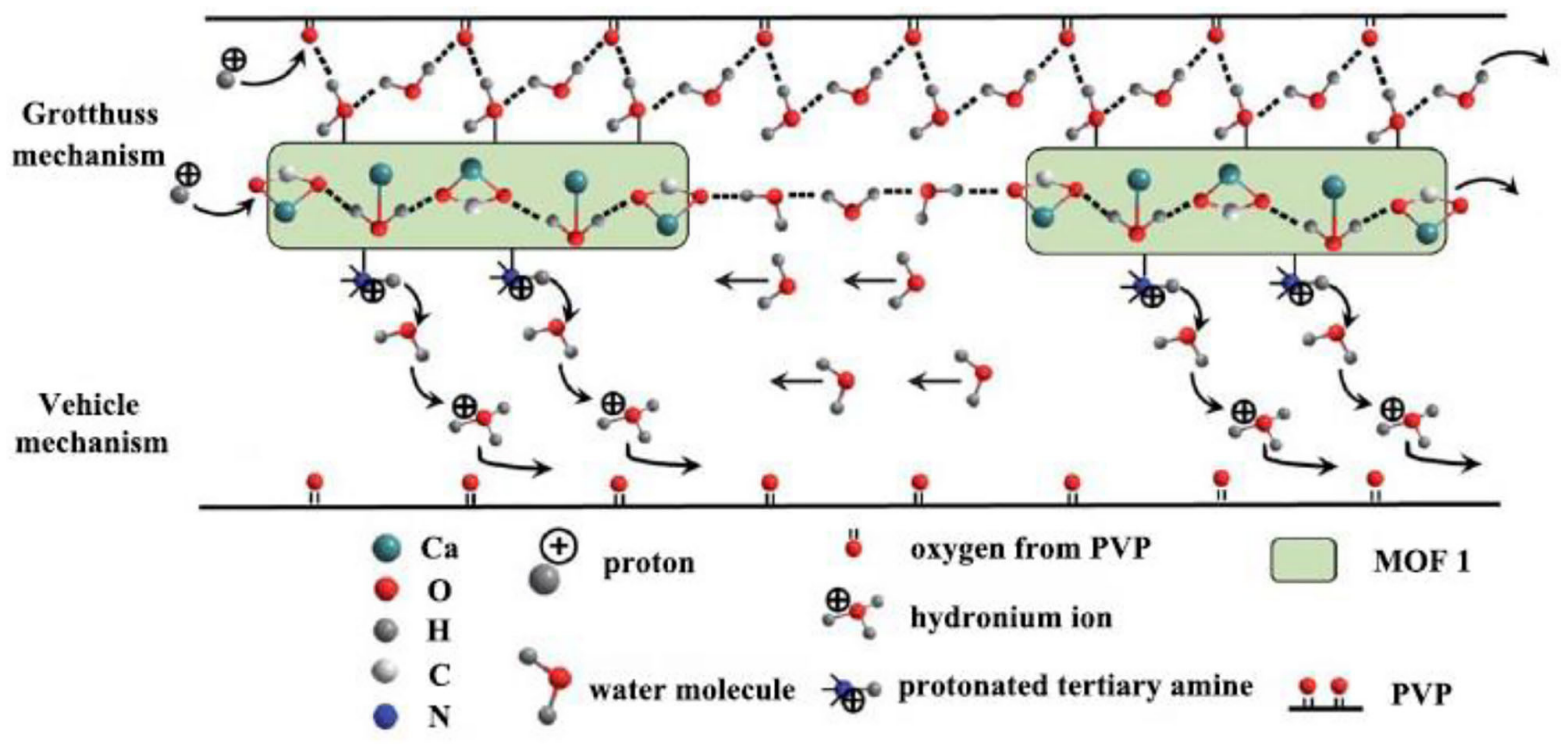
The possible mechanisms of proton transport in the MOF 1–PVP composite membrane. [Readapted with permission from Liang et al. (2013) Copyright 2013, Royal Society of Chemistry].

A chiral 2-D MOF named “MOF 1” was synthesized and embedded into PVP polymer matrix by spin-coating method to prepare composite membrane (Liang et al., 2013). From the Nyquist plot and Arrhenius-type plot, it was found that the proton conductivity of MOF 1 and composite membranes presented a downward trend with the decrease of relative humidity. However, the composite membrane has a significant increase in proton conductivity as the increase of MOF 1 content. The conductivity increased from 4.8 × 10^−7^ S cm^−1^ to 3.2 × 10^−4^ S cm^−1^ with the loading of MOF 1 submicrorods from 3% (MOF 1-PVP-3) to 50 % (MOF 1-PVP-50). It was obvious that the conductivity of MOF 1-PVP-50 was almost one thousand times higher than that of MOF 1-PVP-3. The increase in the conductivity of protons was not only attributed to the capacity of MOF particles to absorb water, but also related to the adsorption and humidification of water by PVP. Figure 11 presented the mechanism of proton transport in the MOF 1–PVP composite membrane.

In recent years, a novel and effective method has been proposed to combine protic ionic liquids (PILs) with porous MOFs. PILs are composed of Brønsted acids and Brønsted bases (Santic et al., 2009; Horike et al., 2012), which as the most promising medium for proton conduction instead of water. PILs have attracted great attention due to their excellent thermal stability, high ionic conductive, low-volatile (Stoimenovski et al., 2010). Nevertheless, the high viscosity of PILs results in unsatisfactory conductivity. It can be seen that the mobility of charges is essentially related to the friction resistance produced by the viscosity of liquids from the Walden rule (Belieres and Angell, 2007). The porous structure of MOFs could disperse PILs sufficiently, which reduced viscosity and saving costs.

## Conclusion

In this paper, we summarized recent advances in improving the performance of PEMs by doping MOFs. MOFs have large specific surface area which can accommodate a large number of water molecules and acidic groups, which providing sufficient receptors for proton transfer. And different structures can be designed according to the needs. However, the high manufacturing cost and unstable performance of purely MOF material crystalline membranes restrict the application in the fuel cells. Preparation of composite membranes by adding MOFs particles into polymers matrix is a simple method. A large amount of hydrogen bonds in MOFs can interact with polymers matrix to build a tighter hydrogen bond network and form additional proton transport channels. The MOFs-modified PEMs have shown outstanding fuel cell performance at elevated temperatures and anhydrous conditions, which has great merit in commercial application.

Different types of MOFs added to the PEMs substrate have different mechanisms for improving composite membrane performance. According to the research and analysis of several typical MOFs materials applied to PEM, it can be seen that UiO-series MOFs have a high-density spatial structure, which endows outstanding stability for water, acid conditions and other solvents. The stability of the composite membranes can be significantly improved by doping UiO-series MOFs to the matrix. MIL-series MOFs in PEMs contain a large number of CUSs, which can provide abundant hydroxyl groups through hydrolysis to promote the conduction of protons in composite membranes. ZIF-series MOFs have high selectivity and good chemical stability in alkaline aqueous solution and other solvents due to their unique hydrophobic pore and surface structure. PEMs containing ZIF-series MOFs can greatly reduce methanol permeability and improve selectivity while maintaining outstanding chemical and thermal stability. In addition, increasing the content of acid groups and mixing several MOFs have exhibited astonishing effect on the improvement of performance.

Many achievements have been made in the research of MOF/PEM composite membranes, which has greatly improved the performance of PEMFCs. However, there still have many challenges in developing MOF/PEM composite membranes:

- select the appropriate MOFs and polymer matrix to improve the performance of composite membranes comprehensively;
- identify the dispersing conditions to ensure that MOFs dispersed uniformly on the polymer matrix;
- optimize the doping concentration of MOFs to obtain the best performance composite membranes;

These aspects directly affect the conductivity, methanol resistance, chemical and thermal stability of the composite membranes, and have an indispensable effect on the improvement of the performance.

More attempts are devoted to preparing high degree of functionalization, preferably both MOFs and new PEMs segments. Thus, more MOF/PEM composite membranes architectures must be designed as new strategies in the rational design of next-generation PEMs materials with high electrochemical PEMFCs performances over a long period of time. Vigorously promoting the development of MOF/PEM composite membranes toward environment-friendly and commercialization.

## Author Contributions

QL: writing-original draft preparation and validation. PZ: funding acquisition, conceptualization, methodology, investigation, and data curation. ZL, DW, ZL, XP, and CL: writing-review and editing. All authors contributed to the article and approved the submitted version.

## Conflict of Interest

The authors declare that the research was conducted in the absence of any commercial or financial relationships that could be construed as a potential conflict of interest.
